# Magnesium stable isotope composition, but not concentration, responds to obesity and early insulin-resistant conditions in minipig

**DOI:** 10.1038/s41598-022-14825-3

**Published:** 2022-06-29

**Authors:** Samuel le Goff, Jean-Philippe Godin, Emmanuelle Albalat, José Manuel Ramos Nieves, Vincent Balter

**Affiliations:** 1 Laboratoire de Géologie de Lyon, ENS de Lyon, Université de Lyon, CNRS, Lyon, France; 2 Nestlé Research, Institute of Food Safety and Analytical Sciences, Lausanne, Switzerland

**Keywords:** Stable isotope analysis, Magnesium, Diabetes

## Abstract

Hypomagnesemia is frequently associated with type 2 diabetes and generally correlates with unfavorable disease progression, but the magnesium status in pre-diabetic conditions remains unclear. Here, the magnesium metabolism is scrutinized in a minipig model of obesity and insulin resistance by measuring variations of the metallome—the set of inorganic elements—and the magnesium stable isotope composition in six organs of lean and obese minipigs raised on normal and Western-type diet, respectively. We found that metallomic variations are most generally insensitive to lean or obese phenotypes. The magnesium stable isotope composition of plasma, liver, kidney, and heart in lean minipigs are significantly heavier than in obese minipigs. For both lean and obese minipigs, the magnesium isotope composition of plasma and liver were negatively correlated to clinical phenotypes and plasma lipoproteins concentration as well as positively correlated to hyperinsulinemic-euglycemic clamp output. Because the magnesium isotope composition was not associated to insulin secretion, our results suggest that it is rather sensitive to whole body insulin sensitivity, opening perspectives to better comprehend the onset of insulin-resistant diabetic conditions.

## Introduction

Magnesium (Mg) has a central role in biology as it is involved in nucleotide binding^1^, enzymatic activity^2^, cell signaling^3^ and cell proliferation^4^. Dysregulation of Mg homeostasis is associated to several pathophysiological conditions, notably diabetes mellitus (DM)^5^. Diabetes mellitus is a group of metabolic diseases characterized by hyperglycemia resulting from defects in insulin secretion and/or insulin action^6^. Type 2 diabetes (T2D) is characterized by a combination of dysfunctional pancreatic β-cells and insulin resistance in target organs^7^. Low Mg^2+^ is a condition associated with T2D through different and non-exclusive mechanisms^8–10^: (1) Mg^2+^ availability is of major importance for glucose metabolism because of its role as adenine nucleotides cofactor^1^; (2) insulin receptors are part of the family of tyrosine kinase receptors, whose function depends on the binding of two Mg^2+^ ions^11^; (3) disturbance of Mg metabolism by medication, notably proton pump inhibitors and thiazide diuretics^12^; (4) mutation of magnesiotropic genes^13^. However, T2D patients do not systematically exhibit hypomagnesemia, except in extreme severity of the disease^14^ where hypomagnesemia is positively associated with hyperglycemia^10^. Oral Mg supplementation appears to improve glycemia and insulin-sensitivity parameters in patients with DM^15^ and with metabolic syndrome^16^.

Another line of evidence indicating that Mg is involved in DM came from the recent observation that the plasma Mg stable isotope composition is lighter in type 1 diabetes (T1D) than in healthy adults, while serum Mg concentration is similar^17^. This finding demonstrates that the DM-related dysregulation of Mg metabolism produces subtle changes in the relative proportions of Mg isotopes at natural abundance. Because these changes, also named isotope fractionation, occur without affecting the overall Mg content, the Mg isotope composition thus appears to be a new and independent means to scrutinize Mg metabolism in health and disease. Isotope fractionation is an infinitesimal physicochemical process, theoretically predicted^18,19^, measurable and reproducible by mass spectrometry which has been used for years in geochemistry to study for instance cosmochemical^20^ or climatological processes^21^. Because vibrational frequencies decrease with mass, heavy isotopes tend, at isotopic equilibrium, to be enriched in the stiffest bonds, in particular those involving the oxidized form and ligands with the stronger electronegativity (O > N > S)^22^. Kinetic processes tend to favor light isotopes, lighter being generally synonymous of faster^19^. In all cases, mass conservation dictates the degree of fractionation that may occur: accumulation of heavy isotopes in one reservoir requires the accumulation of light isotopes in another, so that no fractionation can occur in total reactions that go to completion. The use of metal isotope fractionation has been transformative to the biomarker field for a number of diseases and offers a fresh eye on pathological mechanisms involving metals, such as Cu in cancer^23–25^, hepatic^26,27^ and neurodegenerative^28,29^ diseases, Fe in hemochromatosis^30^ and anemia^31^ and Ca in osteoporosis^32,33^.

Magnesium has three stable isotopes, ^24^Mg, ^25^Mg, and ^26^Mg, with relative abundances of 78.99%, 10.00%, and 11.01%, respectively. The discovery that the Mg isotope composition of chlorophyll-a is fractionated relative to the culture media stimulated interests for the Mg isotopic systematics in biological systems which was further extended to unicellular organisms^34,35^, higher plants^36,37^ and mammals^38,39^. Here, to gain new insights into DM-related dysregulation of Mg metabolism, we have measured the concentration of major (Na, Mg, P, S and K) and minor (Ca, Fe, Cu and Zn) elements and the Mg stable isotope composition (denoted hereafter δ^26^Mg, see “Material and method”) in bone, heart, kidney, liver, muscle and plasma of insulin-resistant and obese (hereafter abbreviated IR-O, n = 9) and lean (n = 6) minipigs along with clinical and molecular phenotypes relevant to glucose metabolism (see “Material and method”). The concentration of minor and major elements will be hereafter denoted the metallome.

## Results

All data generated during this study are included in this published article in Tables S1–S3. Clinical and molecular phenotypes are given in Table S1 and show many significant differences between lean and IR-O minipigs (Fig. S1). Clinical phenotypes (Fig. S1A) encompass morphological, cardiac and adiposity-related traits. Cardiovascular characteristics (systolic and diastolic pressure and heartbeat) are similar in lean and IR-O minipigs (Table S1) and are not shown in Fig. S1. Morphological traits (abdominal circumference, length and body weight) are significantly greater with an almost two-fold change (log2FC = 0.7) in IR-O minipigs, while even greater differences (e.g., log2FC = 5.5 for retroperitoneal fat) are measured for adiposity parameters (backfat thickness, mesenteric fat, omental fat and retroperitoneal fat, Fig. S1A). Among molecular phenotypes, fasting insulin and glucose, triglycerides as well as free fatty acids concentrations are similar in lean and IR-O minipigs (Table S1). IR-O minipigs also show a two-fold increase (log2FC = 1.3, Fig. S1B) in HDL-cholesterol, LDL-cholesterol, total cholesterol and 3-hydroxybutyrate compared to lean animals. The glucose-stimulated insulin secretory capacity (GSIS, measured by hyperglycemic clamp) slightly increases (log2FC = 0.6) in lean versus IR-O minipigs but the difference does not reach the significance level (Wilcoxon test, *P* value = 0.073). The insulin-stimulated systemic glucose utilization (hereafter abbreviated ISGU assessed by hyperinsulinemic-euglycemic clamp) is significantly lower in IR-O by almost half of that observed in lean minipigs (log2FC = − 0.8, Fig. S1C). All these differences indicate a metabolically detrimental phenotype in IR-O minipigs with some features (i.e., visceral adiposity and hypercholesterolemia) commonly observed in metabolic syndrome. The euglycemic-hyperinsulinemic clamp is considered the gold standard to assess whole body insulin sensitivity and the hyperglycemic clamp has proved useful in measuring β-cell function, both techniques being efficient in human^40^ but also in animal models^41^. There is substantial evidence that insulin resistance, typically defined as a decreased of insulin sensitivity, is a precursor of metabolic syndrome and T2D^42^. The presence of some hallmarks of metabolic syndrome along with the significant reduction in insulin sensitivity indicated IR-O animals were at greater risk of reaching a diabetic state than their lean counterparts.

Principal Component Analysis of major and minor elements measured in each tissue and plasma samples (PCA, Fig. S2A) shows that the organ-specific metallomic signatures are stronger than phenotype effects, in other words, there is no metallomic difference (except for plasma P and S) between lean and IR-O phenotypes for a given organ. The organ-specific signature of the metallome has already been described before (Fig. S2B,C) in the mouse^43–45^. The present data show for the first time, however, that eigenvectors and eigenvalues are similarly distributed between mouse and pig, suggesting a highly conserved metallome distribution in mammal organs. Matrix correlations show that elements measured in each organ are most generally positively correlated (Fig. S3), with some exception notably Ca, a pattern already observed in the mouse^45^. Skeletal and cardiac muscle exhibit comparable metallome correlation matrices (Fig. S3B,E), while plasma has a distinctive signature (Fig. S3F).

**Figure 1:**
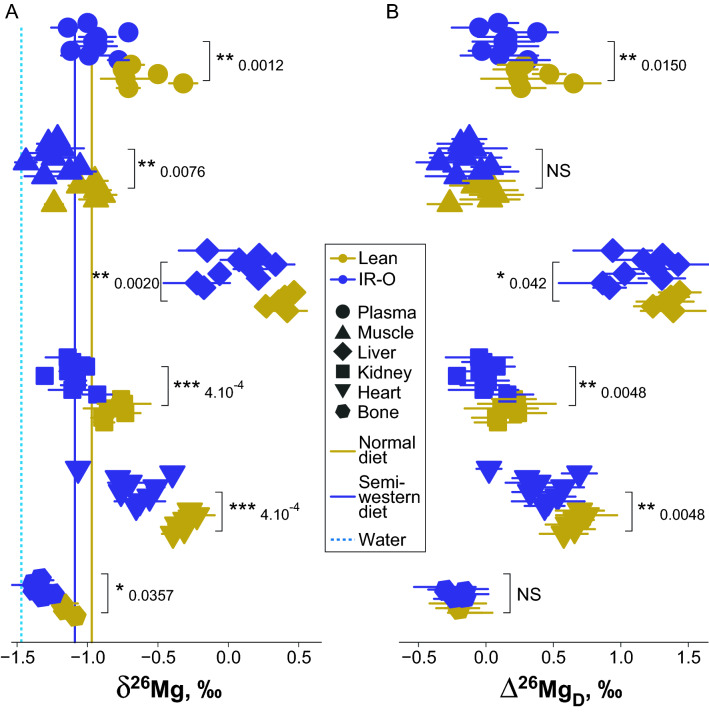
Distribution of the Mg isotope composition in organs on lean and insulin-resistant/obese (IR-O) minipigs. (**A**) δ^26^Mg value in organs, diet and water. (**B**) diet-corrected δ^26^Mg value (∆^26^Mg_D_) in organs. Results of Wilcoxon test *P* value between lean and IR-O minipigs are given. Error bars are two standards deviations of the mean. ***P < 0.001, **P < 0.01, *P < 0.05, NS *P* > 0.05.

During the course of the study, the measured Mg isotope fractionation was mass-dependent (Fig. S4). The δ^26^Mg values range from − 1.5‰ in bone and muscle to + 0.5‰ in liver (Fig. 1A, Table S2). The δ^26^Mg values are organ-specific, and the bodily Mg isotope systematics in minipigs is similar to that described in the mouse^46^, suggesting a conserved pattern among mammals. Noteworthy are the different δ^26^Mg values of skeletal and cardiac muscle, which contrasts with the metallome results. The organ δ^26^Mg values of IR-O minipigs are always significantly lower than those of lean minipigs (Fig. 1A). The δ^26^Mg values of plasma collected 30 and 10 min before hyperinsulinemic-euglycemic clamp are reproducible, exhibiting an average difference of the δ^26^Mg value between the two points of collection of − 0.01‰ and an average absolute value of the difference of the δ^26^Mg value of 0.10‰ (Fig. S5). This natural variability of the plasma Mg isotope composition is three times higher than the external reproducibility of the δ^26^Mg values (~ 0.03‰, n = 85, 2SD), but remains four times lower than the difference between lean and IR-O plasma average δ^26^Mg values (Fig. 1A, Table S2). However, the δ^26^Mg value of semi-Western diet (− 1.09 ± 0.09‰, n = 9, 2SD) offered to IR-O minipigs is slightly lower than that of the normal diet (− 0.97 ± 0.10‰, n = 8, 2SD) offered to lean minipigs, suggesting that part of the observed isotopic differences (~ 0.12‰) between lean and IR-O phenotypes might be due of dietary origin. We therefore calculated the difference between the δ^26^Mg values of organs of lean and obese minipigs and their respective diet (∆^26^Mg_D_). A full mass balance including Mg concentration and Mg isotope composition of diet and water is given in Table S3. The calculation produces unchanged δ^26^Mg values of total Mg intakes for lean and IR-O minipigs. The distribution of the ∆^26^Mg_D_ values in organs is shown in Fig. 1B and yields slightly degraded statistical scores but highlights those significant differences between lean and IR-O minipigs are still conserved except in skeletal muscle and bone. The observed difference of ∆^26^Mg_D_ values between organs suggest that bone is the most ^26^Mg-depleted organ for both phenotypes, whereas liver is the most ^26^Mg-enriched organ. This suggests a preferential incorporation of light Mg isotope during the precipitation of hydroxylapatite, where Mg is known to accumulate into clusters at the rim of the crystallites^48^. In liver, the Mg isotopic fractionation via enzymatic reactions is likely intense and consequential for lipids and glucose regulation. This raises the interest to scrutinize associations at molecular level with surrogate outputs measured in plasma.

**Figure 2:**
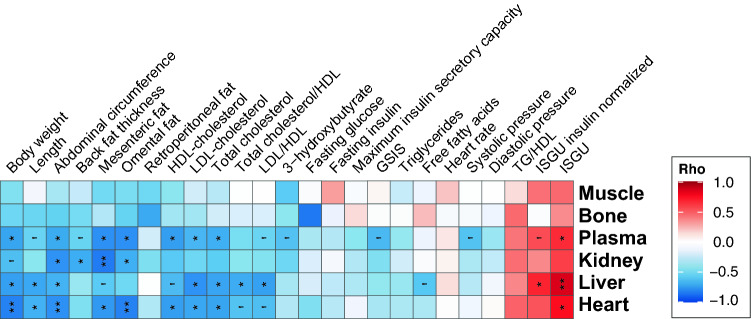
Heatmap of Spearman Rho coefficients of correlations between ∆^26^Mg_D_ values and phenotypes. Adjusted *P* value for multiple testing with the Benjamini & Hochberg correction are given: ***Adj.*P* < 0.001, **Adj.*P* < 0.01, *Adj.*P* < 0.05, ‘Adj.*P* < 0.1.

**Figure 3:**
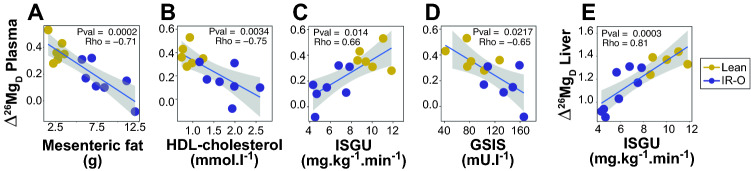
Scatterplots of selected significant correlations discussed in the text. Spearman Rho coefficients and associated P values are given ***P < 0.001, **P < 0.01, *P < 0.05.

To highlight the possible mechanisms at work, we next examine the associations between the metallome and Mg isotope compositions and the clinical and molecular phenotypes by generating heatmaps of Spearman Rho values for each organ (Fig. S6). The metallome does not exhibit many significant associations with phenotypes, except negative correlations between free fatty acids and K and Mg in kidney and Na and Mg in plasma (Fig. S6). In plasma, P is positively and S negatively significantly correlated to several parameters (Fig. S6). Neither the Mg isotope composition nor the metallome is sensitive to cardiovascular parameters. The δ^26^Mg and ∆^26^Mg_D_ values exhibit numerous significant positive and negative correlations with phenotypes that are summarized in Fig. 2 and fully depicted in Fig. S7. On the one hand, ∆^26^Mg_D_ is negatively correlated with adiposity outcomes (fat content) and clinical metabolites (lipoproteins, except triglycerides) phenotypes related to fat metabolism and, on the other hand, positively correlated with ISGU. The significant correlations between ∆^26^Mg_D_ and phenotype values (Spearman Rho coefficient associated with a *P* value < 0.05) are plotted in Fig. S7. Interestingly, the range of phenotypic and Mg isotopic variations are always much more restricted in lean than in IR-O minipigs, highlighting fat-related, glucose-related and Mg disrupted metabolism in IR-O minipigs. Relevant correlations between the plasma ∆^26^Mg_D_ value and phenotypes are shown in Fig. 3. The mesenteric fat content, whether normalized or not to body weight, is well reflected in the plasma ∆^26^Mg_D_ value, with a reduced variability in lean animals (Fig. 3A). This trait can be viewed as a healthy starting point from which visceral adiposity increases as plasma ∆^26^Mg_D_ decreases along with metabolic health in IR-O minipigs. Similar interpretations can also be made for HDL-cholesterol content (Fig. 3B) and other lipoproteins (Fig. S7). The ∆^26^Mg_D_ value is positively correlated to ISGU, with IR-O minipigs having, as expected, poorer insulin sensitivity (Fig. 3C). The ∆^26^Mg_D_ value is also negatively correlated to the GSIS (Fig. 3D). Although greater GSIS in IR-O minipigs might seem contradictory with a diminished metabolic health, it is a clear reflection of the compensatory mechanism to overcome the decrease in ISGU to reach normoglycemia.

## Discussion

Pig, notably the Göttingen strain, is increasingly used as an animal model for obesity and diabetes research (e.g.,^49,50^). Along with the clamp techniques to assess glucose metabolism (insulin sensitivity and insulin secretion), the Göttingen strain fits our purpose to explore the relationship between Mg isotope composition and changes in glucose metabolism since the animals studied here present some of the hallmarks of metabolic syndrome and T2D. In these conditions, the body Mg isotope composition, as typified by the plasma ∆^26^Mg_D_ value, is likely modulated by the glucose metabolism, and not the opposite. Indeed, natural variations of stable isotope composition involve only very subtle changes of the relative abundances of isotopes. For instance, a + 1‰ variation of the δ^26^Mg value corresponds to a deviation of the ^26^Mg/^24^Mg ratio of 10^–4^ (i.e., 100 ppm). Such a minute variation of the Mg isotopic ratio, Mg concentration being independently constant, is unlikely to be the cause of glucose dysregulation, but rather a consequence. This holds for any natural variation of stable isotope composition in the context of biological applications^22–33^. Here, the plasma ∆^26^Mg_D_ value is positively correlated to the utilization of insulin-stimulated systemic glucose (ISGU Fig. 3D), IR-O minipigs having a poorer insulin responsiveness than control minipigs. Also, the plasma ∆^26^Mg_D_ value is correlated to GSIS but not to fasting insulin nor to the maximum insulin secretory capacity (Fig. 2). It is therefore difficult to establish whether the plasma Mg isotope composition is rather controlled by the insulin responsiveness part of the whole glucose metabolism or by its insulin secretion part. Plasma is however a passive circulating reservoir that collects a representative and averaged composition of the body that may be useful for diagnostic purposes, but not a metabolically active organ per se. Interestingly in liver, which is central in glucose metabolism, the ∆^26^Mg_D_ value is strongly correlated with ISGU (Fig. 3E), but not with GSIS (Rho = 0.35, *P* value = 0.27, Fig. S6). This suggest that the Mg isotope composition of liver and, by extension that of plasma, is rather modulated by the insulin responsiveness of glucose than by insulin secretion. In T2D conditions, the control of hepatic glucose production is impaired due to defects in insulin production^51^. Here, both lean and IR-O animals are normoglycemic and their fasting glucose concentration is not correlated to plasma ∆^26^Mg_D_ value (nor to any organ ∆^26^Mg_D_ value, Fig. 2), which suggests that the Mg isotope composition is sensitive in prediabetic conditions involving insulin resistance. This is not the case for Mg concentration, which is insensitive to changes in insulin sensitivity or hyperinsulinemia during pre-diabetes. Our results suggest that Mg metabolism is already disrupted in very early stages of a deteriorating glucose metabolism, which can be detected by changes in isotope compositions only, the concentrations being ultimately modulated during more severe stages of the disease^10,14^. This conclusion suggests that the results obtained by Grigoryan et al.^17^ are not specific to TD1 conditions but rather to phenotypes associated with insulin resistance preceding clinical disease conditions. Measuring the plasma Mg isotope composition therefore opens perspectives to better comprehend the dysregulation of Mg metabolism in pre-diabetic conditions and might pave the road of using the Mg isotope composition as a potential early biomarker of the onset of insulin-resistant prediabetic conditions. This should be challenged by further experiments on the mouse model for which various diabetic mutants exist.

A potentially useful compartment to analyze and overlooked in the present study is urine. The whole-body Mg burden does not vary significantly between obese and lean minipigs because these do not exhibit changes in organ Mg concentration. However, the whole-body Mg isotope composition of obese minipigs is fractionated, being ^26^Mg-depleted or ^24^Mg-enriched, relative to lean minipigs. This isotopic fractionation must be equilibrated by a ^26^Mg-enriched or ^24^Mg-depleted component in obese minipigs to account for mass-balance relative to food isotope composition. This missing component is most probably urine because the major excretory pathway of Mg is through the kidney^52^. The urine Mg isotope composition of diabetic patients or animal models should be therefore higher than that of healthy controls. Testing this hypothesis will strengthen the present proof of concept results obtained on the Göttingen minipig model. A prospective study in adults would be relevant to conduct with baseline and follow-up plasma and urinary Mg isotope determination overtime to confirm the potential utility of this biomarker as a novel and early predictor in diabetes risk assessment.

## Material and method

### Animals and experimental design

The study was performed at Wageningen Research University (the Netherlands), was approved by the Wageningen Research ethical committee (N°AVD 401002017875) and follows guidelines and regulations of the Act on the Animal Testing (The Netherlands) and the recommendations of the ARRIVE guidelines.

After weaning at approximately 10 days of age, female Gottingen minipigs were housed pairwise in pens equipped with an automatic milk replacer dispenser unit. Due to the management complications of rearing both males and females beyond puberty in the experimental setting, as well as the increased risk of developing an adverse metabolic phenotype in adulthood, only female piglets were used in this trial. Twelve and eight minipigs were assigned to the lean and IR-O groups respectively and pen allocation was based on body weight in order to achieved balanced groups. No animals were excluded from the study. The minipigs reported in this report were part of a larger study examining the effect of early nutrition on long-term health. The aim of this manuscript is to report an exploratory result and therefore no sample size calculation was made per se. The exploratory results will be useful for identifying new research directions and for preparing confirmatory statistics. Randomization of feed treatments took place before metabolic testing. Allocation of female piglets to a pen was based on similar body weights for the pairs and an equal mean and standard deviation of their body weights per treatment. Piglets still from different litters were placed in an individual pen and assigned to one of two treatments, IR-O and lean. No analytical randomization was performed for elemental and isotopic analyses. Samples were randomly selected and analyzed over several days. From ~ 10 days to about 11 weeks of age, animals were fully and exclusively fed through the artificial rearing system with controlled amounts of standard milk replacer (Trouw Nutrition Sloten, The Netherlands; Table S4). For two weeks they were fed ad libitum quantities from 07:00 to 17:00 h at which time milk cups were filled with 200 ml of milk replacer for the rest of the evening-night period. From then on milk replacer was offered at 7:00, 12:00 and 17:00 h at a limited rate and sufficient to allow for weight gains comparable to those observed in a reference natural suckling group. At 11 weeks of age, piglets were switched to a solid pelleted diet.

Minipigs in the IR-O group were fed a high-energy, obesogenic diet while the lean animals received a standard SDS minipig diet (Tables S5 and S6). Animals were fed twice per day using controlled amounts of food sufficient to induce an average body weight gain of ~ 1 and ~ 0.4 kg (as recommended per Ellegaard Göttingen Minipigs for adequate development) per week in IR-O and Lean groups, respectively. Only the technical staff looking at the minipigs and the statistical consultant were aware of the minipigs assignment. For the exploratory outcome reported here, the data analyst and statistician that analyzed the data were blinded and only one scientist was aware of the treatments.

### Clamps and tissue collection

At 47 weeks of age, both a hyperinsulinemic-euglycemic (HIEC) and a hyperglycemic clamp (HGC) were performed in sequence in each minipig. Jugular and carotid permanent catheters were inserted surgically 4–7 days to the clamp procedures as described by Koopmans et al.^53^. 1 to 3 days after, systolic, diastolic and mean arterial blood pressure as well as heartbeat were measured using a Hewlett Packard, Model 66S, M1166A (Boeblingen, Germany). Blood pressure and heartbeat were recorded every 30 s for a period of 8 min after a stabilization period of 15- to 30-min of resting behaviour. A heparinized blood sample was taken for the measurement of different metabolites and insulin after the cardiovascular measurements.

Blood samples were taken for measurement of blood glucose concentration at 30 and 10 min before and 10, 20, 30, 40, 50, 60, 70, 80, 90, 100, 110, 120, 135, 150, 165 and 180 min after start of the HIEC. The insulin infusate was prepared using a 5-ml EDTA blood sample collected prior to the procedure and consisted of sterile saline (NaCl), 2% porcine plasma and the pre-estimated amount of human insulin (Actrapid Penfill human insulin, 100 U/ml, Novonordisk). It was infused for 2 min at a rate of 1 mU/kg min after a priming dose of 99 mU/kg min. Also, a variable infusion of a 20% D-glucose solution (Inf eco Braun, 500 ml)) was initiated to maintain blood glucose concentration between 3.5 and 4 mmol/l. HIEC procedures were described previously^54,55^. Baseline endogenous plasma insulin concentration was measured using a porcine insulin ELISA kit (Mercodia, Uppsala, Sweden), and all subsequent insulin values obtained during the steady state phase of the clamp, as induced by exogenously infused human insulin, was measured using a human insulin ELISA kit (Human Insulin kit, IBL International RE53171, Hamburg, Germany).

During the HGC, blood glucose was measured at 30 and 2 min before and 10, 20, 30, 40, 50, 55, 60, 70, 80 and 90 min after the start of the procedure. At t = 0, an infusion of a 20% d-glucose solution (Inf eco Braun, 500 mL)) was started (infusion rate was empirically set at 3 × the body weight in ml/h). Blood glucose concentration was clamped at 10 mmol/l adjusting the infusion rate. At t = 50 an arginine bolus injection (0.8 g/kg body weight) was provided intravenously. Procedures were described previously^54,55^. Plasma insulin concentration was measured on a porcine insulin ELISA kit (Mercodia, Uppsala, Sweden).

At 48 weeks of age, the pigs were euthanized one hour after their daily morning meal. Different organs and tissues were sampled: heart, liver, kidneys, retroperitoneal fat, omental fat, mesenteric fat, as well as the left tibia and gastrocnemius muscle. Samples were collected, flash frozen in liquid nitrogen, and stored at − 80 °C until analyses.

### Sample preparation and Mg stable isotope analysis

Plasma and tissue samples were freeze dried and digested using Milestone Ethos microwave (Milestone, Sorisole, Italy) by a mixture of concentrated distilled HNO_3_ and 30% H_2_O_2._ The ion exchange chromatography procedure and mass spectrometry technique have been extensively described in Ref.^47^. Briefly, Mg was purified using a three steps ion-exchange chromatographic method. The first step (2 ml of AG1-X8 resin) keeps Ca, Mg, P, S, K, Mn and Na with 10 ml of 6 M HCl. The second step removes S, P, Na and Ca by collecting the 18 ml to 42 ml fraction of 1 M HCl passed on 2 ml of AG50W-X12 resin. The third step (210 μl of AG50W-X12 resin) aims at isolating K in 1 M HCl after removing the remaining elements with 13 ml of 0.4 M HCl. Total procedure blank ^24^Mg was typically measured at 4.2 × 10^–3^ V on the Neptune Plus thus representing a 0.01% contribution to an overall Mg signal at 0.5 mg/l. External correction in a sample-standard bracketing approach was applied for instrumental mass bias correction. The intermediate precision, or long-term external reproducibility, that includes inconsistency of sample processing and isotopic ratio measurements, was evaluated from the uncertainties measured on two Mg solutions, namely Cambridge-1 and UKAS, which gives a value of 0.03 ‰ (Cambridge-1: n = 85, 2SD; UKAS: n = 46, 2SD).

The Mg isotope compositions were measured by multi-collectors inductively coupled plasma mass spectrometry (Neptune + Thermo Fisher Scientific, Bremen, Germany) at the Laboratoire de Géologie de Lyon (LGL-TPE). The delta value is given by:

$$\delta {}^{26}Mg=\left[\frac{{\left({}^{26}Mg/{}^{24}Mg\right)}_{Sample}}{{\left({}^{26}Mg/{}^{24}Mg\right)}_{Standard}} -1\right]$$

The DSM3 solution was used as the Mg isotopic standard. Magnesium isotope compositions are given in Table S2. Elemental concentrations were measured by quadrupole inductively coupled plasma mass spectrometry at LGL-TPE using an iCap-Q quadrupole mass spectrometer (Thermo Fisher Scientific, Bremen, Germany). Element concentrations are given in Table S2.

## Supplementary Information

Supplementary Information.
