# Supplementary material for: Magnesium stable isotope composition, but not concentration, responds to obesity and early insulin-resistant conditions in minipig
Source: Sci Rep. 2022 Jun 29;12:10941. doi: 10.1038/s41598-022-14825-3 (PMC9243132; doi:10.1038/s41598-022-14825-3)
Supplement: Supplementary file 1 — Supplementary Information. [file 41598_2022_14825_MOESM1_ESM.pdf]

Supplementary information for

# Magnesium stable isotope composition, but not concentration, responds to obesity and early insulin-resistant conditions in minipigs

Samuel le Goff, Jean-Philippe Godin, Emmanuelle Albalat, José Manuel Ramos Nieves, Vincent Balter

Corresponding author: Vincent.balter@ens-lyon1.fr

This file includes:
Supplementary Figures S1-S7
Supplementary Table S1-S5
Supplementary Reference S1

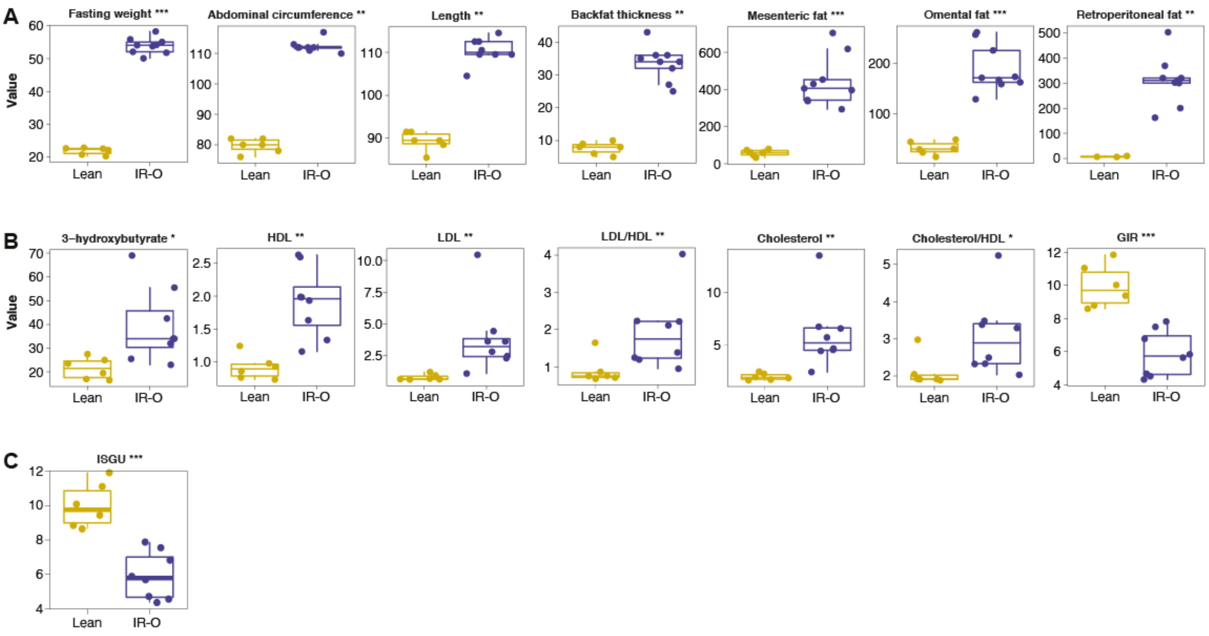


Figure S1: **Boxplots for significantly different phenotypes**. A) Clinical phenotypes. B) Molecular phenotypes. C) Clamp values. ISGU stands for insulin-stimulated systemic glucose utilization. Gold and dark blue stand for lean and IR-O minipigs, respectively. *P* values for Wilcoxon test are given: ***$P < 0.001$, **$P < 0.01$, *$P < 0.05$.

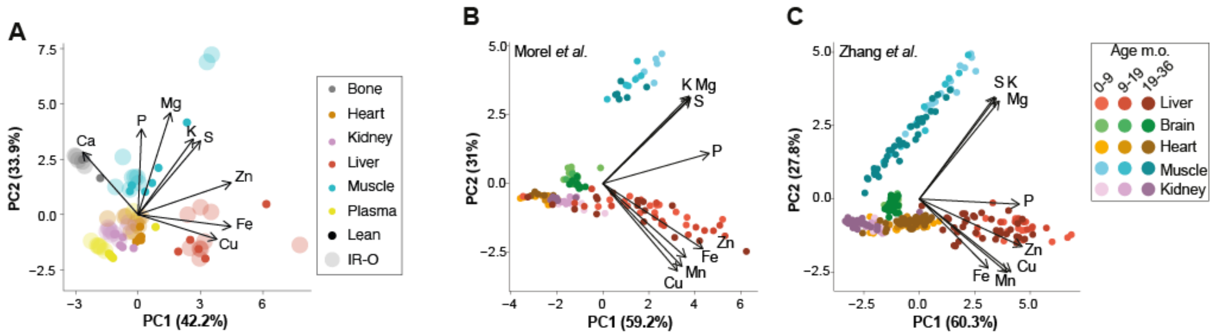


Figure S2: **PCA analysis of the metallome in the minipig and the mouse models**. A) PCA analysis of the present study in minipigs. B) PCA analysis in mice from Morel et al. (45). C) PCA analysis in mice from Zhang et al. (44). Data are normalized to Na for comparison.

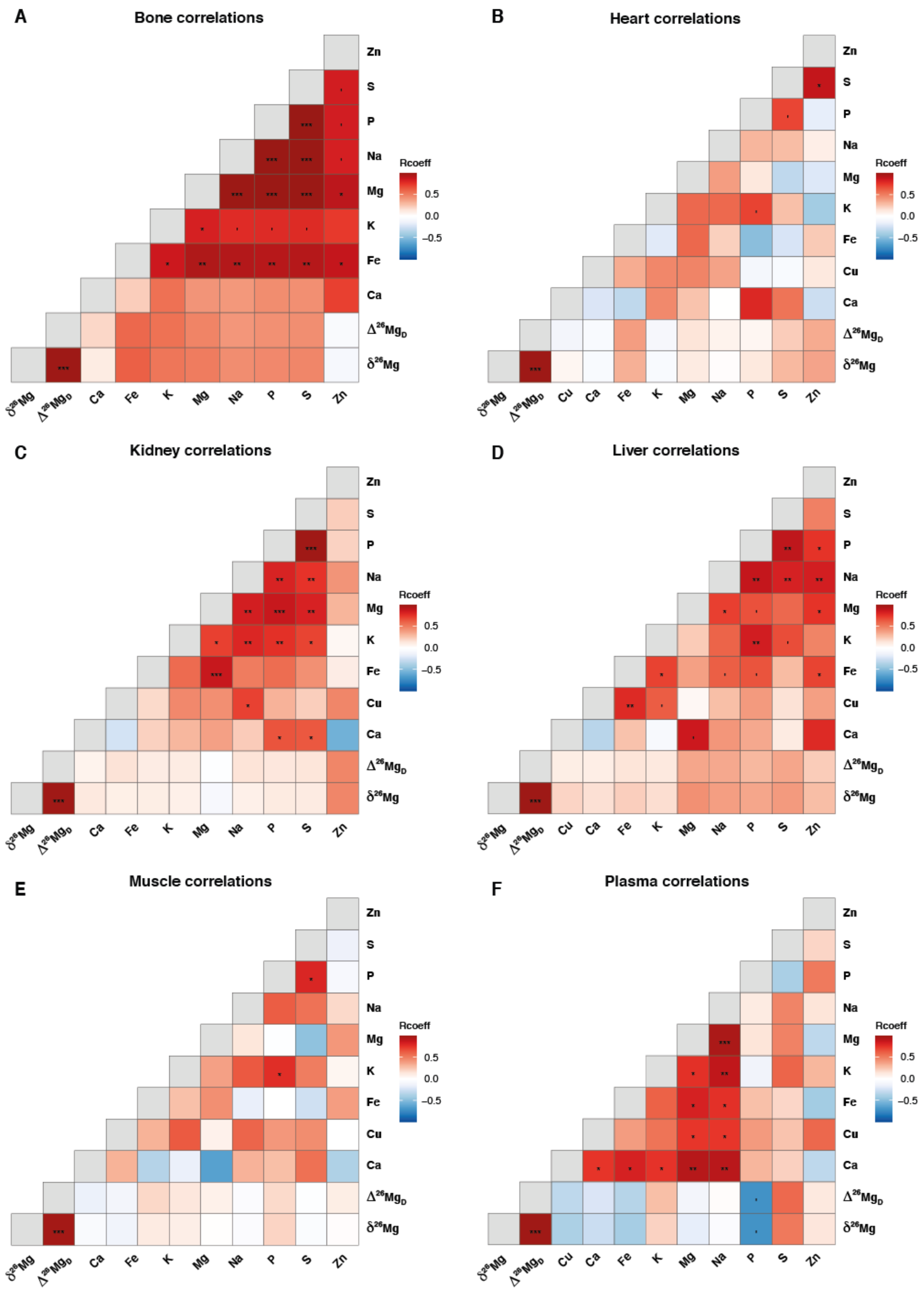


Figure S3: **Heatmap of Spearman Rho coefficients of correlations between the metallome and the Mg isotope compositions in organs**. Adjusted *P* value for multiple testing with the Benjamini & Hochberg correction are given: ***Adj.$P < 0.001$, **Adj.$P < 0.01$, *Adj.$P < 0.05$, ‘Adj.$P < 0.1$.

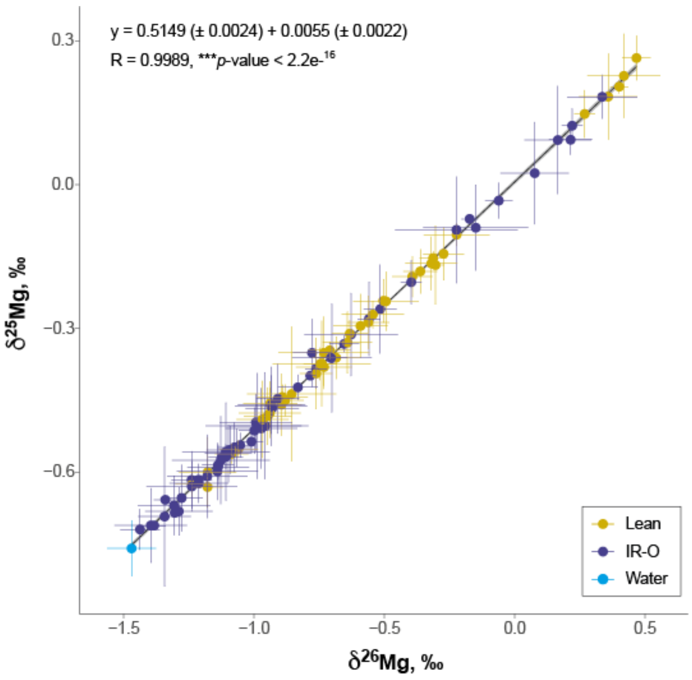


Figure S4: **Mass fractionation in three-isotope space of the results**. The slope of the regression line is 0.515, lying between the theoretical kinetically controlled (0.511) and thermodynamically controlled (0.521) values (ref S1)

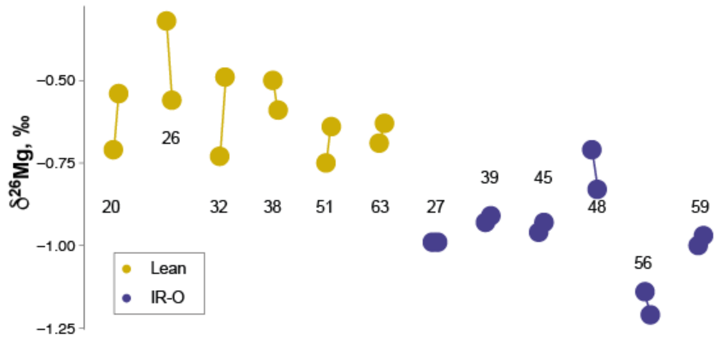


Figure S5: **Dot plot of the values of plasma**. The samples were collected 30 and 10 min before hyperinsulinemic-euglycemic clamp. ID of the animals are indicated.

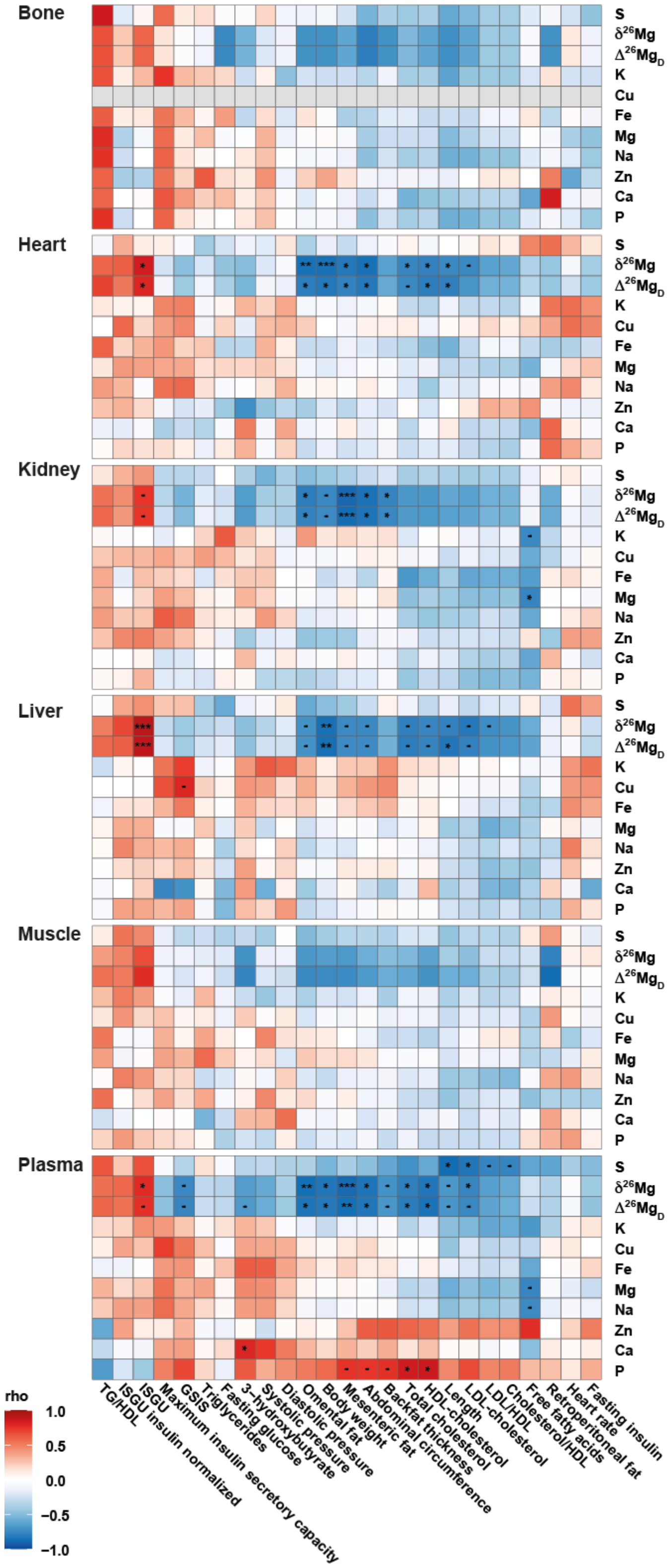


Figure S6: **Heatmap of Spearman Rho coefficients of correlations between the metallome and the Mg isotope compositions, and phenotypes in organs**. Adjusted *P* value for multiple testing with the Benjamini & Hochberg correction are given: ***Adj.*P* < 0.001, **Adj.*P* < 0.01, *Adj.*P* < 0.05, ‘Adj.*P* < 0.1.

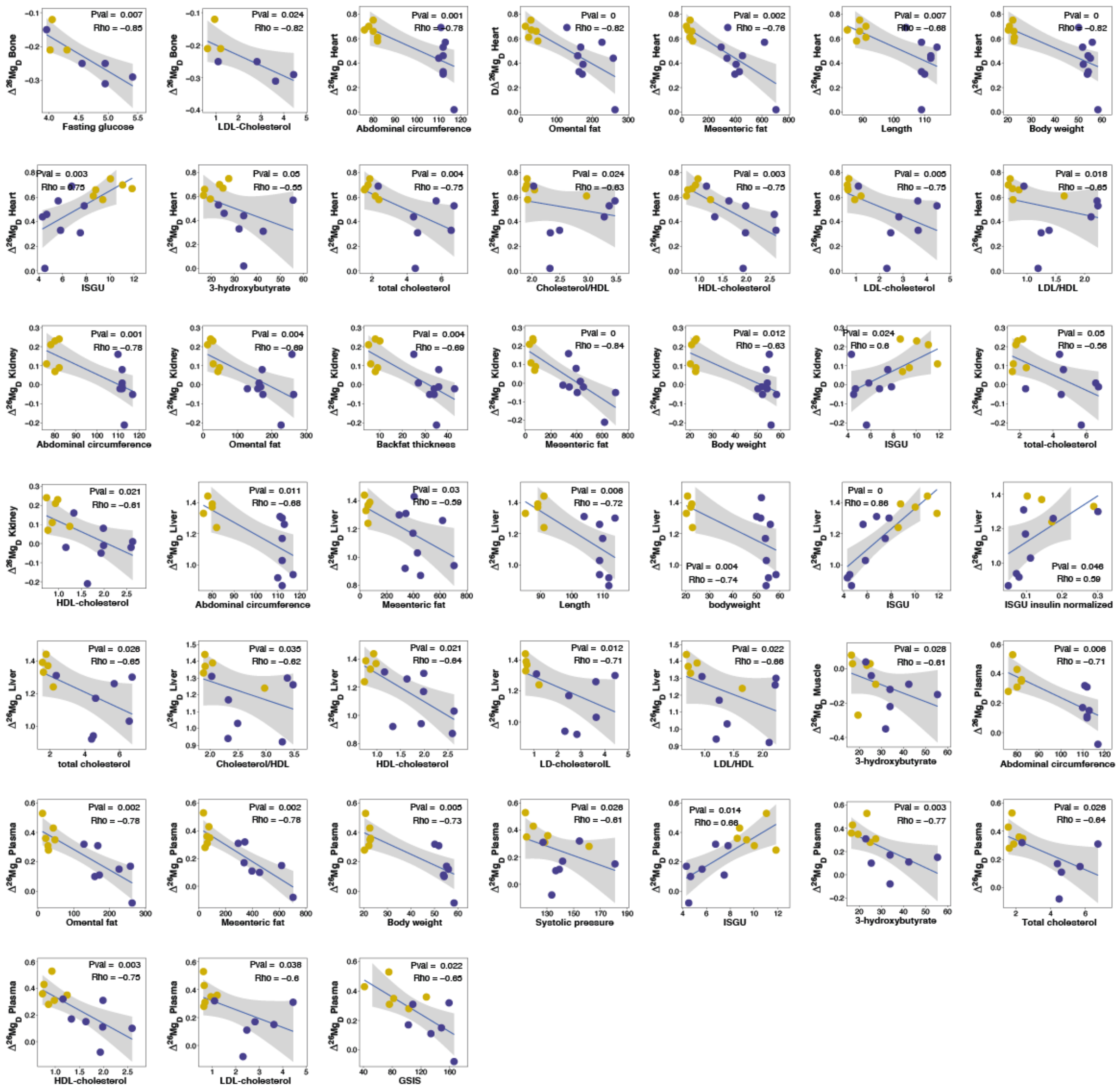


Figure S7: **Scatterplots for significant correlations between the Δ26Mg$_D$ values and phenotypes**. Some correlations are spurious, notably concerning morphological phenotypes (e.g., fasting weight, length or abdominal circumference) because they are driven by only two groups of clustered values. Gold and dark blue stand for lean and IRO minipigs, respectively Spearman Rho coefficients and associated *P* value are given: ***$P < 0.001$, **$P < 0.01$, *$P < 0.05$.

# <u>Table S1</u>: **Phenotypes values of Göttingen minipigs**

| Ind | Status | Abdominal circumference | Omental fat | Retroperitoneal fat | Backfat thickness | Mesenteric fat | Body length | Body weight | Systolic blood pressure | Diastolic blood pressure | Heart rate | Insulin-stimulated systemic glucose utilization (ISGU) | GIR insulin normalized | 3-hydroxybutyrate | Total cholesterol | Total cholesterol:HDL ratio | Free fatty acids | Fasting glucose | Fasting insulin | HDL-cholesterol | LDL-cholesterol | LDL:HDL ratio | Triglycerides | TG:HDL ratio | Glucose-stimulated insulin secretory capacity (GSIS) | Maximum insulin secretory capacity |
|---|---|---|---|---|---|---|---|---|---|---|---|---|---|---|---|---|---|---|---|---|---|---|---|---|---|---|
| 20 | Lean | 82 | 48 | 9.4 | 9 | 80 | 88 | 23 | 115 | 74 | 87 | 9.4 | 0.11 | 20 | 2.395 | 1.92 | 0.50 | 4.1 | 14.0 | 1.25 | 0.95 | 0.76 | 0.35 | 0.28 | 82 | 199 |
| 26 | Lean | 78 | 13 | | 5 | 33 | 91 | 21 | 114 | 101 | 135 | 11.1 | 0.48 | 24 | 1.775 | 1.91 | 0.30 | 4.0 | 16.9 | 0.93 | 0.63 | 0.68 | 0.20 | 0.21 | 75 | 81 |
| 38 | Lean | 80 | 43 | 6.2 | 8 | 74 | 89 | 23 | 120 | 72 | 57 | 8.8 | 0.10 | 17 | 1.565 | 2.05 | 0.20 | 4.7 | 1.2 | 0.77 | 0.66 | 0.86 | 0.35 | 0.45 | 41 | 81 |
| 32 | Lean | 82 | 22 | | 8 | 60 | 91 | 23 | 131 | 87 | 106 | 8.6 | 0.17 | 17 | 2.185 | 2.97 | 0.35 | 4.3 | 17.8 | 0.74 | 1.21 | 1.65 | 0.39 | 0.52 | 127 | 231 |
| 51 | Lean | 76 | 29 | | 6 | 45 | 85 | 20 | 162 | 122 | 102 | 11.9 | 0.29 | 25 | 1.620 | 1.88 | 0.30 | 4.9 | 16.9 | 0.86 | 0.65 | 0.75 | 0.24 | 0.27 | 103 | 202 |
| 63 | Lean | 80 | 28 | 5.7 | 10 | 61 | 89 | 22 | 128 | 84 | 82 | 10.0 | 0.14 | 28 | 1.875 | 1.91 | 0.25 | 4.6 | 9.7 | 0.98 | 0.70 | 0.71 | 0.42 | 0.42 | 76 | 173 |
| 16 | Obese | | 174 | 321 | 32 | 406 | | 52 | | | | | | | | | | | | | | | | | | |
| 22 | Obese | 112 | 167 | 303 | 36 | 294 | 114 | 52 | 127 | 93 | 115 | 7.8 | 0.30 | 23 | 6.720 | 3.38 | 0.65 | 5.4 | 21.0 | 1.99 | 4.44 | 2.23 | 0.24 | 0.12 | 109 | 128 |
| 27 | Obese | 112 | 159 | 163 | 34 | 453 | 112 | 54 | 137 | 94 | 78 | 4.7 | 0.05 | 26 | 13.590 | 5.24 | 0.80 | 4.0 | | 2.60 | 10.45 | 4.03 | 0.36 | 0.14 | | |
| 34 | Obese | 112 | 162 | 370 | 27 | 430 | 109 | 54 | 114 | 77 | 75 | 5.8 | 0.11 | 32 | 6.555 | 2.49 | 0.35 | 5.0 | 12.8 | 2.63 | 3.64 | 1.38 | 0.40 | 0.15 | 71 | 98 |
| 39 | Obese | 110 | 258 | 321 | 25 | 338 | 112 | 55 | 142 | 113 | 106 | 4.3 | 0.08 | 34 | 4.390 | 3.29 | 0.45 | 5.0 | 15.6 | 1.34 | 2.82 | 2.11 | 0.28 | 0.21 | 102 | 133 |
| 45 | Obese | 113 | 226 | 311 | 35 | 618 | 109 | 56 | 181 | 128 | 85 | 5.6 | 0.18 | 56 | 5.690 | 3.48 | 0.25 | 4.5 | 7.7 | 1.64 | 3.63 | 2.22 | 0.43 | 0.26 | 148 | 245 |
| 48 | Obese | 111 | 129 | 504 | 43 | 343 | 104 | 50 | 154 | 108 | 113 | 6.8 | 0.09 | 69 | 2.355 | 2.03 | 0.35 | 4.6 | 20.2 | 1.16 | 1.10 | 0.95 | 0.20 | 0.17 | 159 | 321 |
| 56 | Obese | 117 | 263 | 301 | 34 | 704 | 109 | 58 | 134 | 94 | 108 | 4.5 | 0.07 | 34 | 4.490 | 2.32 | 0.30 | 5.2 | 19.5 | 1.94 | 2.31 | 1.19 | 0.45 | 0.23 | 166 | 228 |
| 59 | Obese | 112 | 171 | 201 | 36 | 397 | 110 | 54 | 139 | 89 | 109 | 7.5 | 0.10 | 43 | 4.620 | 2.33 | 0.30 | 4.8 | 24.5 | 1.99 | 2.48 | 1.25 | 0.24 | 0.12 | 134 | 216 |

Table S2: **Mg isotope composition and metallomic values of Göttingen minipigs**

| Ind | Status | Organ | $\delta^{26}Mg$ | ±2SD | $\delta^{25}Mg$ | ±2SD | n | Ca | Cu | Fe | K | Mg | Na | P | S | Zn |
|---|---|---|---|---|---|---|---|---|---|---|---|---|---|---|---|---|
| 20 | Lean | Bone | -1.09 | 0.06 | -0.56 | 0.02 | 3 | 1118 |  | 11 | 148 | 1992 | 2391 | 37781 | 580 | 273 |
| 26 | Lean | Bone | -1.18 | 0.11 | -0.60 | 0.07 | 3 | 1045 |  | 2 | 51 | 763 | 991 | 17237 | 262 | 26 |
| 32 | Lean | Bone | -1.18 | 0.06 | -0.63 | 0.04 | 3 | 1262 |  | 4 | 113 | 1155 | 1292 | 21920 | 334 | 41 |
| 22 | Obese | Bone | -1.38 | 0.04 | -0.71 | 0.01 | 2 | 897 |  | 3 | 67 | 734 | 941 | 15361 | 233 | 29 |
| 27 | Obese | Bone | -1.24 | 0.08 | -0.62 | 0.06 | 2 | 881 |  | 2 | 50 | 784 | 979 | 15896 | 244 | 30 |
| 34 | Obese | Bone | -1.40 | 0.14 | -0.71 | 0.08 | 2 | 1176 |  | 2 | 79 | 910 | 1198 | 19834 | 304 | 35 |
| 39 | Obese | Bone | -1.34 | 0.04 | -0.66 | 0.01 | 2 | 1127 |  | 4 | 69 | 1446 | 1793 | 29145 | 443 | 41 |
| 48 | Obese | Bone | -1.34 | 0.10 | -0.69 | 0.15 | 2 | 1196 |  | 3 | 83 | 1030 | 1348 | 22037 | 330 | 33 |
| 20 | Lean | Heart | -0.39 | 0.08 | -0.19 | 0.04 | 5 | 14 | 27 | 333 | 13731 | 1037 | 3673 | 8964 | 9057 | 111 |
| 26 | Lean | Heart | -0.27 | 0.08 | -0.14 | 0.05 | 5 | 1036 | 24 | 257 | 15750 | 1230 | 3709 | 11840 | 9653 |  |
| 32 | Lean | Heart | -0.36 | 0.06 | -0.18 | 0.05 | 4 | 19 | 28 | 333 | 16558 | 1230 | 4930 | 10971 | 10195 | 119 |
| 38 | Lean | Heart | -0.31 | 0.05 | -0.15 | 0.01 | 3 | 97 | 18 | 338 | 11796 | 1017 | 3490 | 8947 | 8196 | 87 |
| 51 | Lean | Heart | -0.30 | 0.03 | -0.17 | 0.08 | 2 |  | 28 | 394 | 15445 | 1381 | 3603 | 9920 | 8271 | 33 |
| 63 | Lean | Heart | -0.22 | 0.13 | -0.10 | 0.02 | 2 | 21 | 23 | 285 | 15719 | 1263 | 3564 | 10080 | 7411 | 15 |
| 16 | Obese | Heart | -0.70 | 0.15 | -0.36 | 0.11 | 2 |  | 36 | 526 | 16099 | 2518 | 3685 | 9954 | 8007 | 1722 |
| 22 | Obese | Heart | -0.56 | 0.04 | -0.28 | 0.08 | 5 | 17 | 28 | 284 | 14096 | 1063 | 3614 | 9404 | 9380 | 107 |
| 27 | Obese | Heart | -0.63 | 0.18 | -0.31 | 0.09 | 3 | 94 | 19 | 323 | 12439 | 975 | 2324 | 8770 | 8243 | 84 |
| 34 | Obese | Heart | -0.76 | 0.04 | -0.38 | 0.04 | 5 | 609 | 19 | 221 | 15073 | 1163 | 3487 | 12022 | 10135 |  |
| 39 | Obese | Heart | -0.65 | 0.06 | -0.33 | 0.02 | 3 | 96 | 9 | 161 | 5583 | 470 | 923 | 4312 | 4079 | 43 |
| 45 | Obese | Heart | -0.52 | 0.06 | -0.26 | 0.09 | 2 |  | 28 | 367 | 14983 | 1255 | 3986 | 9511 | 8788 | 67 |
| 48 | Obese | Heart | -0.40 | 0.04 | -0.20 | 0.05 | 5 | 698 | 23 | 291 | 17200 | 1038 | 3773 | 12948 | 10616 |  |
| 56 | Obese | Heart | -1.07 | 0.00 | -0.55 | 0.04 | 2 |  | 24 | 318 | 15917 | 1258 | 4502 | 9800 | 7403 | 12 |
| 59 | Obese | Heart | -0.78 | 0.02 | -0.40 | 0.00 | 2 |  | 26 | 250 | 15293 | 1289 | 3084 | 9674 | 7521 | 25 |
| 20 | Lean | Kidney | -0.88 | 0.07 | -0.45 | 0.03 | 5 | 23 | 63 | 254 | 10524 | 846 | 5025 | 10401 | 7592 | 119 |
| 26 | Lean | Kidney | -0.76 | 0.06 | -0.39 | 0.07 | 5 | 1179 | 46 | 282 | 11225 | 960 | 6873 | 16144 | 10171 |  |
| 32 | Lean | Kidney | -0.73 | 0.11 | -0.38 | 0.04 | 5 | 26 | 103 | 261 | 9942 | 933 | 8543 | 12154 | 8393 | 149 |
| 38 | Lean | Kidney | -0.90 | 0.08 | -0.46 | 0.07 | 3 | 977 | 66 | 338 | 14498 | 1186 | 7607 | 16638 | 11170 | 30 |
| 51 | Lean | Kidney | -0.86 | 0.17 | -0.44 | 0.14 | 2 | 177 | 139 | 294 | 11574 | 1019 | 9149 | 12138 | 7507 | 88 |
| 63 | Lean | Kidney | -0.74 | 0.19 | -0.37 | 0.09 | 3 | 141 | 192 | 334 | 12210 | 1077 | 7598 | 12305 | 7501 | 131 |
| 16 | Obese | Kidney | -1.14 | 0.06 | -0.58 | 0.04 | 2 | 174 | 56 | 363 | 11989 | 1057 | 6003 | 11591 | 7387 | 65 |
| 22 | Obese | Kidney | -1.10 | 0.09 | -0.56 | 0.06 | 5 | 24 | 87 | 224 | 11617 | 826 | 6476 | 11762 | 8705 | 128 |
| 27 | Obese | Kidney | -1.11 | 0.17 | -0.56 | 0.10 | 3 | 105 | 29 | 172 | 3007 | 329 | 1274 | 4099 | 3299 | 45 |
| 34 | Obese | Kidney | -1.08 | 0.07 | -0.55 | 0.05 | 5 | 726 | 89 | 200 | 11563 | 887 | 6745 | 15009 | 9987 | 38 |
| 39 | Obese | Kidney | -0.93 | 0.10 | -0.47 | 0.02 | 2 | 84 | 14 | 49 | 1451 | 189 | 838 | 2484 | 1785 | 29 |
| 45 | Obese | Kidney | -1.30 |  | -0.68 |  | 1 | 255 | 136 | 258 | 12180 | 1068 | 8225 | 11596 | 7482 | 41 |
| 48 | Obese | Kidney | -1.11 | 0.07 | -0.57 | 0.03 | 5 | 608 | 61 | 416 | 11100 | 1611 | 7773 | 16542 | 10532 | 410 |
| 56 | Obese | Kidney | -1.14 | 0.16 | -0.60 | 0.06 | 2 | 307 | 126 | 297 | 12455 | 1125 | 8485 | 12334 | 7550 | 108 |
| 59 | Obese | Kidney | -1.01 | 0.04 | -0.54 | 0.02 | 2 | 145 | 161 | 325 | 13116 | 1036 | 8007 | 12382 | 7382 | 86 |
| 26 | Lean | Liver | 0.47 | 0.05 | 0.26 | 0.05 | 4 | 442 | 28 | 1465 | 8770 | 2310 | 3744 | 14240 | 8723 | 801 |
| 32 | Lean | Liver | 0.27 | 0.04 | 0.15 | 0.05 | 4 | 17 | 35 | 818 | 7674 | 699 | 2591 | 10592 | 7720 | 268 |
| 38 | Lean | Liver | 0.42 | 0.14 | 0.23 | 0.09 | 2 | 94 | 19 | 767 | 4791 | 424 | 598 | 6770 | 4708 | 172 |
| 51 | Lean | Liver | 0.36 | 0.11 | 0.18 | 0.09 | 3 |  | 31 | 969 | 10399 | 817 | 2253 | 11374 | 6618 | 391 |
| 63 | Lean | Liver | 0.40 | 0.03 | 0.20 | 0.01 | 2 |  | 47 | 1708 | 8514 | 1258 | 3555 | 12013 | 7041 | 893 |
| 16 | Obese | Liver | 0.34 | 0.13 | 0.18 | 0.05 | 2 |  | 84 | 1704 | 9258 | 814 | 1244 | 9969 | 5196 | 400 |
| 22 | Obese | Liver | 0.21 | 0.08 | 0.09 | 0.03 | 4 | 15 | 38 | 1362 | 9087 | 602 | 2365 | 10280 | 7005 | 317 |
| 27 | Obese | Liver | -0.22 | 0.24 | -0.09 | 0.11 | 3 | 104 | 23 | 844 | 9097 | 636 | 1388 | 10850 | 6937 | 363 |
| 34 | Obese | Liver | -0.06 | 0.05 | -0.03 | 0.04 | 3 | 322 | 17 | 621 | 4274 | 955 | 1329 | 6959 | 3884 | 345 |
| 39 | Obese | Liver | -0.17 | 0.03 | -0.07 | 0.01 | 2 | 91 | 18 | 749 | 4801 | 376 | 781 | 6600 | 4121 | 147 |
| 45 | Obese | Liver | 0.17 | 0.13 | 0.09 | 0.11 | 2 |  | 51 | 1472 | 9750 | 832 | 2821 | 12252 | 6619 | 654 |
| 48 | Obese | Liver | 0.22 | 0.04 | 0.12 | 0.04 | 4 | 52166 | 46 | 875 | 33921 | 7656 | 9786 | 30878 | 8247 | 403 |
| 56 | Obese | Liver | -0.15 | 0.20 | -0.09 | 0.09 | 2 |  | 61 | 1740 | 9332 | 846 | 2578 | 10680 | 6015 | 664 |
| 59 | Obese | Liver | 0.08 | 0.13 | 0.02 | 0.11 | 2 |  | 54 | 1740 | 10992 | 779 | 2607 | 12167 | 6673 | 430 |
| 20 | Lean | Muscle | -1.24 | 0.06 | -0.62 | 0.03 | 3 | 17 | 8 | 79 | 15286 | 1143 | 1942 | 8614 | 9845 | 195 |
| 26 | Lean | Muscle | -0.95 | 0.09 | -0.48 | 0.06 | 5 | 506 | 14 | 67 | 16169 | 901 | 2036 | 9308 | 10854 | 105 |
| 32 | Lean | Muscle | -0.89 | 0.10 | -0.44 | 0.03 | 4 | 13 | 14 | 192 | 17160 | 1169 | 1556 | 8684 | 8514 | 124 |
| 38 | Lean | Muscle | -0.94 | 0.12 | -0.48 | 0.05 | 2 | 97 | 5 | 109 | 12365 | 965 | 738 | 6974 | 8027 | 175 |
| 51 | Lean | Muscle | -0.94 | 0.10 | -0.46 | 0.05 | 2 | 132 | 13 | 137 | 15077 | 1155 | 1865 | 7457 | 8745 | 244 |
| 63 | Lean | Muscle | -1.06 | 0.07 | -0.55 | 0.02 | 2 | 3 | 9 | 356 | 15564 | 1325 | 1334 | 7431 | 7572 | 179 |
| 16 | Obese | Muscle | -1.29 | 0.13 | -0.68 | 0.05 | 3 | 68 | 9 | 150 | 15504 | 1256 | 1787 | 8161 | 8469 | 239 |
| 22 | Obese | Muscle | -1.05 | 0.05 | -0.54 | 0.03 | 5 | 12 | 5 | 32 | 14275 | 943 | 1661 | 7489 | 8040 | 106 |
| 27 | Obese | Muscle | -1.13 | 0.19 | -0.57 | 0.09 | 3 | 99 | 4 | 94 | 12937 | 965 | 515 | 7336 | 7751 | 132 |
| 34 | Obese | Muscle | -1.44 | 0.08 | -0.72 | 0.04 | 5 | 810 | 18 | 102 | 16639 | 1016 | 1692 | 10384 | 10346 | 89 |
| 39 | Obese | Muscle | -1.31 | 0.14 | -0.67 | 0.06 | 3 | 92 | 6 | 133 | 12594 | 967 | 490 | 7019 | 7347 | 133 |
| 45 | Obese | Muscle | -1.24 | 0.17 | -0.63 | 0.05 | 3 | 73 | 15 | 127 | 14994 | 1071 | 1816 | 7678 | 8492 | 283 |
| 48 | Obese | Muscle | -1.28 | 0.08 | -0.65 | 0.07 | 5 | 677 | 12 | 76 | 13042 | 737 | 1941 | 7688 | 9262 | 111 |
| 56 | Obese | Muscle | -1.21 | 0.04 | -0.62 | 0.00 | 2 | 62 | 8 | 98 | 15179 | 1429 | 1736 | 7956 | 7536 | 170 |
| 59 | Obese | Muscle | -1.18 | 0.15 | -0.61 | 0.09 | 4 | 72 | 14 | 118 | 13697 | 1347 | 1506 | 6916 | 6291 | 139 |

## Table S2: **continued**

| Ind | Status | Organ | $\delta^{26}Mg$ | ±2SD | $\delta^{25}Mg$ | ±2SD | n | Ca | Cu | Fe | K | Mg | Na | P | S | Zn |
|---|---|---|---|---|---|---|---|---|---|---|---|---|---|---|---|---|
| 20 | Lean | plasma-1 | -0.71 | 0.08 | -0.35 | 0.02 | 5 | 51 | 38 | 17 | 19820 | 210 | 32559 | 1329 | 10072 | 13 |
| 20 | Lean | plasma-2 | -0.54 | 0.12 | -0.27 | 0.03 | 3 | 46 | 38 | 18 | 19744 | 214 | 34044 | 1364 | 10043 | 14 |
| 26 | Lean | plasma-1 | -0.32 | 0.10 | -0.16 | 0.06 | 4 | | | | | | | | | |
| 26 | Lean | plasma-2 | -0.56 | 0.08 | -0.29 | 0.06 | 3 | | | | | | | | | |
| 32 | Lean | plasma-1 | -0.73 | 0.17 | -0.35 | 0.08 | 3 | 50 | 34 | 28 | 19515 | 200 | 34468 | 1094 | 9491 | 13 |
| 32 | Lean | plasma-2 | -0.49 | 0.13 | -0.24 | 0.06 | 3 | 47 | 32 | 25 | 20288 | 196 | 34924 | 1135 | 9529 | 14 |
| 38 | Lean | plasma-1 | -0.50 | 0.02 | -0.24 | 0.05 | 3 | 58 | 28 | 38 | 20253 | 260 | 37028 | 1078 | 11255 | 13 |
| 38 | Lean | plasma-2 | -0.59 | 0.10 | -0.30 | 0.07 | 3 | 53 | 25 | 37 | 19669 | 237 | 33829 | 945 | 10170 | 13 |
| 51 | Lean | plasma-1 | -0.75 | 0.06 | -0.37 | 0.03 | 4 | 1349 | 46 | 46 | 23394 | 318 | 40641 | 1219 | 10191 | |
| 51 | Lean | plasma-2 | -0.64 | 0.09 | -0.33 | 0.06 | 5 | 1384 | 44 | 44 | 23086 | 311 | 42520 | 1258 | 10302 | |
| 63 | Lean | plasma-1 | -0.69 | 0.09 | -0.36 | 0.04 | 3 | 1280 | 34 | 42 | 22592 | 282 | 38989 | 1277 | 9895 | |
| 63 | Lean | plasma-2 | -0.63 | 0.07 | -0.31 | 0.03 | 2 | 1387 | 35 | 40 | 22692 | 293 | 41189 | 1267 | 9747 | |
| 22 | Obese | plasma-1 | -0.78 | 0.07 | -0.35 | 0.07 | 3 | 51 | 32 | 19 | 17764 | 165 | 31043 | 1396 | 9353 | 14 |
| 27 | Obese | plasma-1 | -0.99 | 0.20 | -0.50 | 0.11 | 2 | 70 | 34 | 34 | 16381 | 190 | 30996 | 2036 | 9213 | 14 |
| 27 | Obese | plasma-2 | -0.99 | 0.14 | -0.50 | 0.06 | 2 | 49 | 27 | 34 | 16180 | 185 | 29818 | 1966 | 8929 | 14 |
| 34 | Obese | plasma-1 | -1.12 | 0.01 | -0.57 | 0.02 | 2 | | | | | | | | | |
| 39 | Obese | plasma-1 | -0.93 | 0.14 | -0.46 | 0.08 | 3 | 57 | 24 | 27 | 13775 | 176 | 27246 | 1019 | 7691 | 11 |
| 39 | Obese | plasma-2 | -0.91 | 0.08 | -0.45 | 0.07 | 3 | 56 | 29 | 35 | 17945 | 211 | 32491 | 1237 | 9851 | 11 |
| 45 | Obese | plasma-1 | -0.96 | 0.14 | -0.50 | 0.11 | 4 | 1376 | 44 | 37 | 19757 | 296 | 41435 | 1826 | 9494 | |
| 45 | Obese | plasma-2 | -0.93 | 0.13 | -0.46 | 0.06 | 3 | 1357 | 52 | 47 | 20208 | 340 | 42032 | 1827 | 9680 | |
| 48 | Obese | plasma-1 | -0.71 | 0.06 | -0.36 | 0.01 | 2 | | | | | | | | | |
| 48 | Obese | plasma-2 | -0.83 | 0.08 | -0.42 | 0.03 | 2 | | | | | | | | | |
| 56 | Obese | plasma-1 | -1.14 | 0.12 | -0.59 | 0.03 | 5 | 1288 | 43 | 45 | 20162 | 307 | 37801 | 1573 | 9836 | |
| 56 | Obese | plasma-2 | -1.21 | 0.11 | -0.62 | 0.04 | 6 | 1300 | 41 | 41 | 20400 | 273 | 37732 | 1572 | 9892 | |
| 59 | Obese | plasma-1 | -1.00 | 0.06 | -0.51 | 0.05 | 3 | 1421 | 37 | 57 | 23736 | 284 | 41266 | 1672 | 9676 | |
| 59 | Obese | plasma-2 | -0.97 | 0.14 | -0.51 | 0.08 | 3 | 1390 | 36 | 46 | 23152 | 278 | 40543 | 1604 | 9325 | |

Table S3: **Calculation of the whole diet Mg isotope composition**. The calculation includes Mg isotope composition and concentration in solid food and water and assumes that the water/food mass ratio for Göttingen pigs is 2.5.

**Mean feed intake pellets (g/day/cage/2 pigs)**

| | | | Days | | | | | | | | | | | | | | | | | | | | | | |
|---|---|---|---|---|---|---|---|---|---|---|---|---|---|---|---|---|---|---|---|---|---|---|---|---|---|
| **Cage** | **Batch nr** | **Treatment** | **73-86** | **87-100** | **101-114** | **115-128** | **129-142** | **143-156** | **157-170** | **171-184** | **185-198** | **199-212** | **213-226** | **227-240** | **241-254** | **255-268** | **269-282** | **283-296** | **297-310** | **311-324** | **325-331** | **Total food intake (kg)** | **Total water intake(kg)** | **Total food Mg intake (g) *** | **Total water Mg intake (g) #** | **$\delta^{26}$Mg intake (‰) *#¥** |
| 10 | 1 | Obese | 500 | 600 | 943 | 1000 | 1000 | 1000 | 1000 | 1000 | 1343 | 1400 | 1400 | 1400 | 1400 | 1400 | 1500 | 1600 | 1600 | 1600 | 1600 | 11.6 | 29.1 | 19.9 | 1.3 | -1.08 |
| 15 | 2 | Obese | 500 | 600 | 829 | 1000 | 1000 | 1000 | 1000 | 1000 | 1343 | 1400 | 1400 | 1400 | 1400 | 1400 | 1500 | 1600 | 1600 | 1600 | 1600 | 11.6 | 29.0 | 19.8 | 1.3 | -1.08 |
| 20 | 2 | Obese | 500 | 600 | 829 | 1000 | 1000 | 1000 | 1000 | 1000 | 1343 | 1400 | 1400 | 1400 | 1400 | 1400 | 1500 | 1600 | 1600 | 1600 | 1600 | 11.6 | 29.0 | 19.8 | 1.3 | -1.08 |
| 25 | 3 | Obese | 500 | 600 | 914 | 1000 | 1000 | 1000 | 1000 | 1000 | 1343 | 1400 | 1400 | 1400 | 1400 | 1400 | 1500 | 1600 | 1600 | 1600 | 1600 | 11.6 | 29.1 | 19.9 | 1.3 | -1.08 |
| 28 | 3 | Obese | 750 | 900 | 1371 | 1500 | 1500 | 1500 | 1500 | 1321 | 1343 | 1400 | 1400 | 1400 | 1400 | 1400 | 1500 | 1600 | 1600 | 1600 | 1600 | 13.3 | 33.2 | 22.7 | 1.5 | -1.08 |
| 5 | 1 | Obese | 500 | 600 | 943 | 1000 | 1000 | 1000 | 1000 | 1000 | 1343 | 1400 | 1400 | 1400 | 1400 | 1400 | 1500 | 1600 | 1600 | 1600 | 1600 | 11.6 | 29.1 | 19.9 | 1.3 | -1.08 |
| 3 | 1 | Lean | 400 | 400 | 400 | 457 | 500 | 600 | 600 | 600 | 686 | 700 | 700 | 700 | 750 | 800 | 850 | 900 | 900 | 900 | 900 | 6.4 | 15.9 | 15.2 | 0.7 | -0.96 |
| 12 | 2 | Lean | 400 | 400 | 400 | 457 | 500 | 600 | 600 | 600 | 686 | 700 | 700 | 700 | 750 | 800 | 850 | 900 | 900 | 900 | 900 | 6.4 | 15.9 | 15.2 | 0.7 | -0.96 |
| 19 | 2 | Lean | 400 | 400 | 400 | 457 | 500 | 600 | 600 | 600 | 686 | 700 | 700 | 700 | 750 | 800 | 850 | 900 | 900 | 900 | 900 | 6.4 | 15.9 | 15.2 | 0.7 | -0.96 |
| 32 | 3 | Lean | 600 | 600 | 600 | 675 | 750 | 900 | 900 | 793 | 686 | 700 | 700 | 700 | 750 | 800 | 850 | 900 | 900 | 900 | 900 | 7.3 | 18.3 | 17.4 | 0.8 | -0.96 |

* with a concentration of 2381 ppm and 1710 ppm for normal and semi-western diets, respectively.

# with a concentration of 44 ppm in water.

¥ with a $\delta^{26}$Mg value of -0.97‰, -1.09‰ and -1.44‰ for normal diet, semi-western diet and water, repectively.

Table S4: **Diet formulation of standard milk replacer minipig diet.**

| Ingredient | Control milk replacer (Lactose) |
|---|---|
| WPC-75%CP | 29.40% |
| Lactose | 24.05% |
| Vana Grasa 80C (coconut/malto) | 18.28% |
| Vana-Grasa 80D (palmoil/whey) | 18.23% |
| Maltodextrin 20 M500 | 2.45% |
| Di-Potassium phosphate 17.7%P | 1.47% |
| Citric acid Anh West | 0.98% |
| Sodium chloride | 0.98% |
| Calcium carbonate Pwd FineWhite | 0.74% |
| SLOTEN 1942 PREMIX | 0.49% |
| Calcium formate 98% Fine | 0.49% |
| Calcium acetate 99% OAB | 0.49% |
| Magnesium sulphate Anh 20% | 0.49% |
| Emulsifier E487 silica | 0.34% |
| Potassium sorbate | 0.33% |
| DL-methionine 99% West | 0.31% |
| L-Lysine HCl 98% | 0.18% |
| L-Threonine 98% | 0.08% |
| Silica Tix-O-Sil 38 | 0.06% |
| L-Tryptophan 98% | 0.06% |
| Sweetener | 0.03% |
| Vanilla Aroma | 0.03% |
| Vit E 50% Adsorbate | 0.02% |
| Iron sulphate 1aq 30% min Fine | 0.02% |
| Copper sulphate 5aq 25% Fine | 0.01% |
| | 0.00% |
| Total | 100% |

Table S5: **Diet formulation of high-energy, obesogenic diet (SK2_2) and control, lean SDS minipig diet.**

| | SK2_2<br>0.5% cholesterol<br>% | SDS SMP(E)<br>Standard Mini Pig Diet |
|---|---|---|
| Barley | 10.25 | * |
| Wheat | 8.00 | * |
| Soja hulls RC 320-360 | 35.93 | * |
| Potato protein | 3.69 | |
| Wheat gluten protein | 5.16 | |
| Sucrose | 20.00 | |
| Lard | 8.50 | |
| Trans-fat soja oil | 4.00 | |
| Cholesterol | 0.50 | |
| Limestone, CaCO3 (powder) | 1.35 | |
| Mono-Calcium phosphate | 1.79 | |
| NaCl | 0.62 | |
| Premix 2 g/kg | 0.20 | |
| L-Tryptophan | 0.01 | |
| **Total** | **100.00** | |
| | | |
| Oat hulls and bran | | * |
| Wheatfeed | | * |
| De-hulled extracted toasted soya | | * |
| Molasses | | * |
| Sunflower extracted | | * |
| Macro minerals | | * |
| Micro minerals | | * |
| Vitamins | | * |

Table S6: **Nutrient composition of the high-energy, obesogenic diet (OB) and control, lean SDS Minipig diet**

| | | OB<br>0.5% cholesterol | SDS SMP(E)<br>Standard Mini Pig Diet |
|---|---|---|---|
| Dry matter | g | 925.7 | 900 |
| Crude ash | g | 58.9 | 73.5 |
| Crude protein | g | 130.0 | 130.3 |
| Crude fat | g | 137.6 | 21.3 |
| C18:2 | g | 12.5 | 3.1 |
| Crude fiber | g | 127.6 | 145.2 |
| Digestible crude oil | g | | 12.0 |
| Digestible crude protein | g | | 94.1 |
| Starch (amylase method) | g | 102.4 | 271.2 |
| Sugars | g | 222.9 | 55.4 |
| Non-starch polysaccharides | g | 279.8 | |
| Nitrogen free extract | g | | 524.1 |
| Total dietary fibre | g | | 296.6 |
| EW | - | 1.10 | |
| Gross energy | MJ | | 13.8 |
| Digestible energy | MJ | | 11.43 |
| Metabolisable energy | MJ | | 10.98 |
| Net energy diet | MJ | 9.70 | |
| Calcium | g | 10.1 | 10.1 |
| Phosphorus | g | 5.3 | 5.9 |
| Digestible phosphorus (faeces) | g | 3.7 | 3.7 |
| Inositol-phosphate | g | 0.6 | |
| Ca/digestible phosphorus | | 2.7 | |
| Magnesium | g | 1.1 | 3.1 |
| Sodium | g | 2.6 | 2.9 |
| Potassium | g | 6.0 | 13.7 |
| Chloride | g | 4.2 | 4.2 |
| Electrolyte balance | meq | 150 | |
| Iron | mg | 376 | 161.59 |
| Copper | mg | 25 | 17.73 |
| Zinc | mg | 94 | 118.08 |
| Cobalt | µg | | 59.60 |
| Iodine | µg | | 62.68 |
| Selenium | µg | | 211.35 |
| Fluorine | mg | | 16.58 |
| LYS | g | 6.3 | 6.3 |
| MET | g | 2.2 | 1.8 |
| CYS | g | 2.5 | 2.1 |
| M+C | g | 4.7 | 3.9 |
| THR | g | 4.9 | 4.9 |
| TRP | g | 1.6 | 1.6 |
| ILE | g | 5.4 | 5.3 |
| ARG | g | 6.1 | 8.8 |
| PHE | g | 6.7 | 6.2 |
| HIS | g | 3.0 | 3.3 |
| LEU | g | 9.9 | 9.2 |
| TYR | g | 5.5 | 3.7 |
| VAL | g | 6.4 | 6.4 |
| ALA | g | 5.1 | 0.6 |
| ASP | g | 10.1 | 5.07 |
| GLU | g | 27.2 | 18.1 |
| GLY | g | 6.7 | 10.0 |
| PRO | g | 10.6 | 7.4 |
| SER | g | 6.7 | 4.3 |
| Cumulative AA | g | 126.8 | |
| Ileal digestible LYS | g | 4.6 | |
| Ileal digestible MET | g | 1.9 | |
| Ileal digestible CYS | g | 1.9 | |
| Ileal digestible M+C | g | 3.7 | |
| Ileal digestible THR | g | 3.6 | |
| Ileal digestible TRP | g | 1.2 | |
| Ileal digestible ILE | g | 4.4 | |
| Ileal digestible ARG | g | 5.3 | |
| Ileal digestible PHE | g | 5.7 | |
| Ileal digestible HIS | g | 2.3 | |
| Ileal digestible LEU | g | 8.3 | |
| Ileal digestible TYR | g | 4.4 | |
| Ileal digestible VAL | g | 5.0 | |

Supplementary reference:

S1 E. D. Young, A. Galy, H. Nagahara, Kinetic and equilibrium mass-dependent isotope fractionation laws in nature and their geochemical and cosmochemical significance. *Geochim Cosmochim Acta*. **66**, 1095-1104 (2002).
